# Preparation of a *Klebsiella pneumoniae* conjugate nanovaccine using glycol-engineered *Escherichia coli*

**DOI:** 10.1186/s12934-023-02099-x

**Published:** 2023-05-06

**Authors:** Yan Liu, Chao Pan, Kangfeng Wang, Yan Guo, YanGe Sun, Xiang Li, Peng Sun, Jun Wu, Hengliang Wang, Li Zhu

**Affiliations:** 1 State Key Laboratory of Pathogen and Biosecurity, Beijing Institute of Biotechnology, Beijing, 100071 China; 2 College of Life Science, Hebei University, Baoding, 071002 China

**Keywords:** Glycosyltransferase, Bioconjugate vaccine, Conjugate nanovaccine, *Klebsiella pneumoniae*, Bacterial chassis

## Abstract

**Background:**

Engineered strains of *Escherichia coli* have been used to produce bioconjugate vaccines using Protein Glycan Coupling Technology (PGCT). Nanovaccines have also entered the vaccine development arena with advances in nanotechnology and have been significantly developed, but chassis cells for conjugate nanovaccines have not been reported.

**Results:**

To facilitate nanovaccine preparation, a generic recombinant protein (SpyCather4573) was used as the acceptor protein for O-linked glycosyltransferase PglL, and a glycol-engineered *Escherichia coli* strain with these two key components (SC4573 and PglL) integrated in its genome was developed in this study. The targeted glycoproteins with antigenic polysaccharides produced by our bacterial chassis can be spontaneously bound to proteinous nanocarriers with surface exposed SpyTag in vitro to form conjugate nanovaccines. To improve the yields of the targeted glycoprotein, a series of gene cluster deletion experiments was carried out, and the results showed that the deletion of the *yfdGHI* gene cluster increased the expression of glycoproteins. Using the updated system, to the best of our knowledge, we report for the first time the successful preparation of an effective *Klebsiella pneumoniae* O1 conjugate nanovaccine (KPO1-VLP), with antibody titers between 4 and 5 (Log10) after triple immunization and up to 100% protection against virulent strain challenge.

**Conclusions:**

Our results define a convenient and reliable framework for bacterial glycoprotein vaccine preparation that is flexible and versatile, and the genomic stability of the engineered chassis cells promises a wide range of applications for biosynthetic glycobiology research.

**Supplementary Information:**

The online version contains supplementary material available at 10.1186/s12934-023-02099-x.

## Background

In recent years, as bacterial protein glycosylation systems continue to be elucidated, a method known as Protein Glycan Coupling Technology (PGCT) [1] to produce glycoproteins has emerged as a promising field of research. Three key components are required to produce glycoproteins with site-specifical modifications of the desired glycan structures [2–4]: the glycosyltransferase, the glycan synthesis gene cluster, and the acceptor protein. The method is based on the biocatalytic coupling of polysaccharides to recombinantly expressed proteins using a glycosyltransferase in a bacterial host cell (usually *E. coli*). Depending on the glycosyltransferase used, there are two main types of glycoproteins, those based on protein *N*-glycosylation systems (e.g., *N*-oligosaccharyltransferase PglB in *Campylobacter jejuni* [3, 5, 6]) and those based on protein O-glycosylation systems (e.g., O-oligosaccharyltransferase PglL in *Neisseria meningitidis* [7, 8] and O-oligosaccharyltransferase PglS in *Acinetobacter baylyi* [9, 10]). The main advantage of PGCTs over chemical cross-linking methods is that in vivo coupling can be done in one step to produce a glycoprotein, which can be further purified to obtain a relatively homogeneous end product without the cumbersome process of purifying the carrier protein, the polysaccharide, and the cross-linked end product, with fewer downstream purification steps and without the need for multiple quality control procedures within the process of purifications.

In addition to coordinating the expression of acceptor proteins, polysaccharides, and glycosyltransferases to improve production efficiency, the selection of suitable engineered host cells is crucial. The development of microbial genome editing technology (such as CRISPR-Cas9 tools) provides the basis for constructing microbial cell factories with improved biomanufacturing efficiencies [11]. *E. coli*, as a model strain for the study of microbial genetics, physiology, and metabolism, is one of the important chassis cells due to its well-understood genetic background.

Using *E. coli* as a bacterial chassis for PGCT dates back as far as 1976 when Neuhard and Thomassen et al*.* constructed strain SO874 with the deletion of the O-antigens (also known as O-specific polysaccharides or OPS) and capsular polysaccharide (CPS) synthesis gene clusters [12]. In 1999, Liu and Reeves et al*.* found that W3110 was naturally deficient in O-antigen due to the inactivation of the *wbbL* gene in the O-antigen synthesis cluster by an IS5 insertional mutation [13]. In 2005, Feldman et al*.* constructed strain CLM24, derived from W3110, which is deficient in the O-antigen ligase WaaL which transfers the glycan to the lipid A core, competing with oligosaccharyltransferase (OST) for the lipid-linked oligosaccharide (LLO) substrate [14]. In the same year, Linton et al*.* constructed strain CLM37, which is deficient in WecA which initiates the first biosynthetic step involved in the synthesis pathway of O-antigens and the enterobacterial common antigen (ECA) synthesis pathway in *E. coli* W3110 [15]. The deletion of WecA precludes the transfer of GlcNAc to Und-P, thereby preventing the assembly of ECA and *E. coli* endogenous O-antigen from GlcNAc residues, ultimately favoring the assembly of heterologously expressed glycosomes with their own glycans. In 2006, Wacker et al*.* constructed an O-antigen deficient strain lacking the O-antigen polymerase Wzy [16]. In the same year, Alaimo et al*.* constructed an ECA gene cluster deletion strain based on strain SO874 [17]. In 2008, Perez et al*.* deleted the *waaL* gene based on strain SO874, and in 2014, Musumeci et al*.* [18] deleted the *waaL* gene and *wecA* gene based on strain SO874 [19]. The above engineered *E. coli* strains have partially excluded endogenous glycan interference, allowing for a wide range of applications in PGCT.

As PGCT requires three key components, two or three expression plasmids are typically required for producing glycoproteins. Plasmid-based expression systems have many advantages, such as high expression based on high copy numbers of plasmids. However, plasmids can be unstable and lost from the cell after continuous replication [20]. In addition, multi-copy plasmids significantly alter normal host cell metabolism, and this metabolic burden can interfere in plasmid replication [21] and the translation of plasmid-encoded genes [22]. Integrating PGCT elements into chromosomes would not only reduce their metabolic burden on the host but may also increase glycoprotein production [23]. Therefore, to circumvent these plasmid-related problems, many groups have reported chromosomal integration of multigene pathways to produce heterologous metabolites [19–23].

In 2016, Van den Dobbelsteen et al*.* integrated the heterologous O antigen gene cluster ExPEC onto the genome, replacing the own *rfb* gene cluster in *E. coli* [24], which demonstrated the feasibility of genomic integration. In 2018, Herbert et al*.* integrated the glycosyltransferase PglB derived from *Campylobacter jejuni* onto the genome [25]. In the same year, Strutton et al*.* integrated PglB onto the CLM24 genome and used the strong constitutive promoter BBa_J23119 to control its expression [26]. Their works greatly promoted the development of chassis cells. In 2019, Yates et al., designed a series of exquisite orthogonal tests, in which they integrated the *pgL* minimal motif and/or glycosyltransferase PglB into the MG1655 genome, replacing the endogenous O-antigen gene cluster (*glf-gnd*) and/or the ECA gene cluster, using the promoter of the *galF* gene or promoter BBa_J23110 to control expression. The yields of glycoproteins by the double integrant strain were comparable or higher compared to the classic plasmid-based system. Moreover, only one plasmid encoding the acceptor protein was required, improving the genetic stability of the system and simplifying the process of producing the desired glycoprotein [27].

Like the field of conjugate vaccines against bacterial infection that has been extensively researched, nanovaccines have also entered the vaccine development arena with advances in nanotechnology and have been developed significantly. Nanovaccines commonly refer to a new generation of vaccines that use nanoparticles as transport carriers and/or adjuvants for disease prevention or treatment. Because they are similar in size to microbial pathogens, nanovaccines have high antigen loading rates and good stability in the body to ensure that antigens are not prematurely degraded, and thus they are better able to stimulate the immune system to trigger cellular and humoral immune responses [28, 29]. Several nanovaccines have been successfully introduced into the clinic, such as Recombivax HB and Engerix-B for hepatitis B [30]. Our laboratory has previously developed a Nano-B5 platform for preparing self-assembled nanovaccines, including conjugate nanovaccines, which has a stronger preventive effect against infection than traditional conjugated vaccines [31]. However, chassis design for conjugate nanovaccines has not been reported until now. In this study, we used the glycosyltransferase PglL, paired with a recombinant SpyCatcher acceptor protein (SpyCatcher4573, or SC4573) derived from the SpyCatcher/SpyTag system, to construct a well-designed glyco-recorded chassis strain for the development of nanovaccines. In particular, the gene encoding the acceptor protein (SC4573) and glycosyltransferase (PglL) were integrated into the chromosome to reduce the cellular metabolic burden and allow for easier antigenic glycan replacement, allowing for rapid production of various nanovaccines according to actual needs.

*Klebsiella pneumoniae* (*K. pneumoniae*) is the second most infectious conditional pathogen after *E. coli*. In particular, the emergence of carbapenem-resistant strains (CRKP) in the last two decades has further exacerbated the pressure on clinical antimicrobial therapy. The rapid global spread of CRKP has been associated with an increase in lethality [32–34], which has led it to be classified as a human health threat emergency [35]. Although vaccines against *K. pneumoniae* are being developed, there is still no approved vaccine for clinical use. A recombinant conjugate vaccine prepared by enzymatically linking the CPS of serotypes K1 and K2 to the carrier protein EPA in glycosyl-engineered *E. coli* cells has been reported to be protective against *K. pneumoniae* infection [36, 37]. Compared to the wide variety of K serotypes (related to CPS), the O serotypes (related to OPS) are less diverse, and the O1 and O2 serotypes account for more than 60% of the ultra-broad spectrum β-lactamase resistant and carbapenem-resistant strains [38]. Therefore, in this study, we selected the OPS of *K. pneumoniae* serotypes O1 and O2 as glycol-antigens for producing conjugate nanovaccines. The results showed that mice immunized with *K. pneumoniae* O1 conjugate nanovaccine (KPO1-VLP) produced by our glycosyl-engineered *E. coli* chassis achieved 100% survival under the challenge with the toxic O1 serotype strain.

## Results

### The influence of endogenous glycan synthesis pathways on targeted glycoprotein yields

**Fig. 1:**
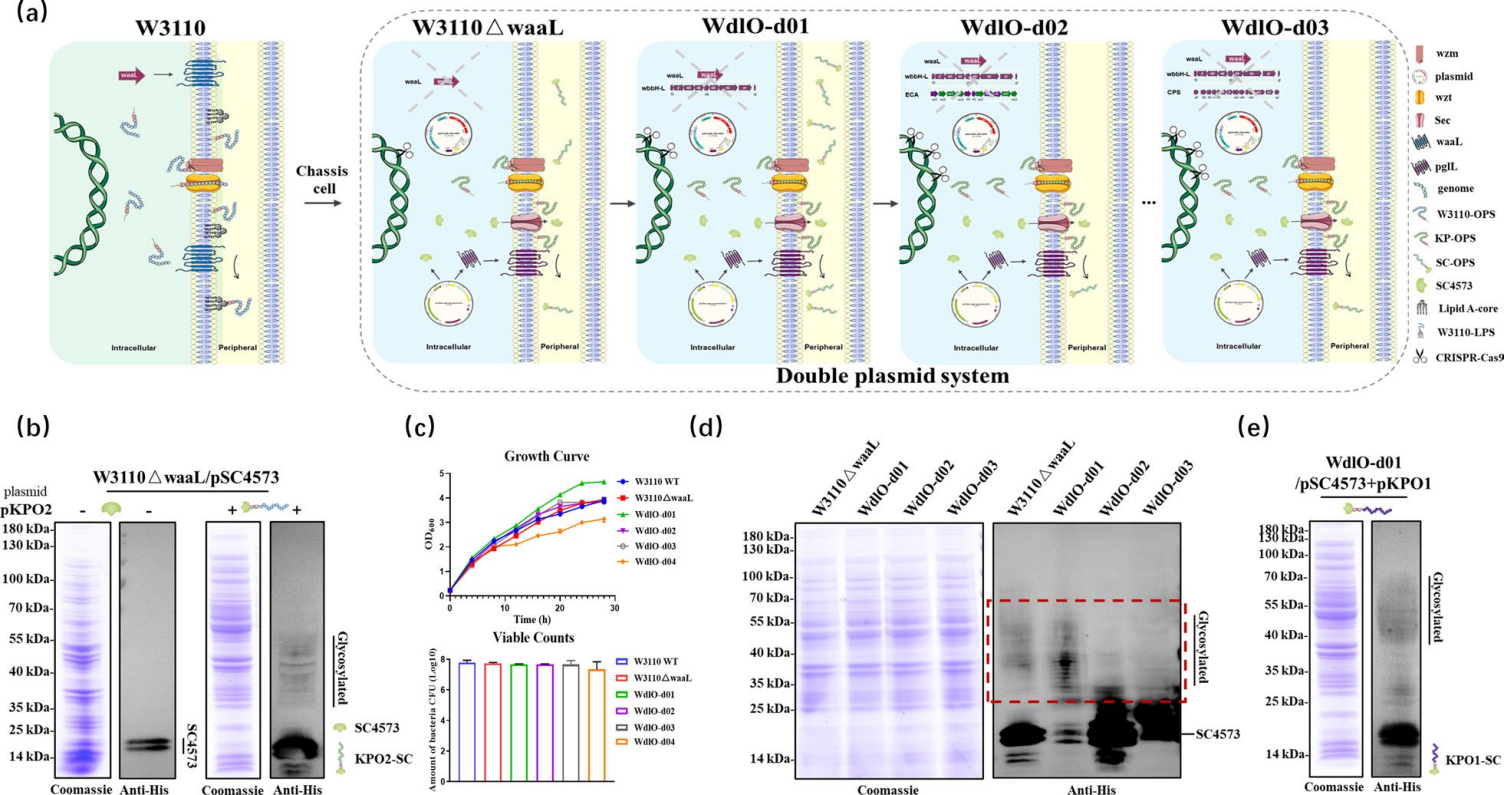
Heterologous glycoprotein expression in different* E. coli* mutants. **a** Schematic representation of a series of gene cluster scarless deletion mutants derived from *E. coli* wild-type strain W3110. **b** Detection of the expressions of acceptor protein SC4573 and glycoprotein KPO2-SC in W3110 ∆*waaL*. **c** Growth curves and viable counts of *E. coli* wild-type strains and each mutant strain (W3110 ∆*waaL*, WdlO-d01, WdlO-d02, WdlO-d03, and WdlO-d04). Each strain was inoculated with a seed solution at OD_600_ = 2.0, and LB ratio of 1:100; the viable bacteria were counted by gradient dilution-coated LB plates at OD_600_ = 2.0. **d** Comparison of KPO2-SC glycoprotein expression in each mutant strain. **e** Detection of KPO1-SC glycoprotein expression in mutant strain WdlO-d01

A good *E. coli* chassis for PCGT requires excluding competition from endogenous glycan synthesis pathway to facilitate the coupling of exogenous antigenic glycans to acceptor proteins to produce targeted glycoproteins. Therefore, we used CRISPR-Cas9 tools to construct a series of gene clusters (including the *wbbH–L* gene cluster, the ECA gene cluster, and the CPS gene cluster) scarless deletion mutants derived from *E. coli* wild-type strain W3110 (Fig. 1a). First, a basic chassis strain W3110 ∆*waaL* with the deletion of the gene *waaL* that encoded the O-antigen ligase were obtained using the pCas/pTargetF system described by Jiang et al*.* [39]. Then, two plasmids encoding the acceptor protein SC4573 (pET28a-SpyCatcher4573, or pSC4573) and the *K. pneumoniae* O2 serotype antigenic polysaccharide (pACYC184-355OPS, or pKPO2) were electroporated into the basic chassis at the same time. The expression of the acceptor protein SC4573 and the targeted glycoprotein KPO2-SpyCatcher4573 (KPO2-SC) were detected by SDS-PAGE and WB using anti-His antibodies (Fig. 1b). Based on this, to increase the yield and reduce the metabolic burden on the chassis cells, we successively deleted three gene clusters and obtained the following mutants: W3110 ∆*waaL*∆*wbbH–L* (renamed WdlO-d01), W3110 ∆*waaL*∆*wbbH–L*∆*ECA* (renamed WdlO-d02), W3110 ∆*waaL*∆*wbbH–L*∆*CPS* (renamed WdlO-d03), and W3110 ∆*waaL*∆*wbbH–L*∆*ECA*∆*CPS* (renamed WdlO-d04). All the gene deletion mutants were verified by sequencing (Additional file 1: Fig. S1). Growth rates were assessed for each deletion strain. Although there was no significant difference in the number of viable bacteria, cell growth rates decreased abruptly after consecutive deletions (WdlO-d04 in Fig. 1c). Then, two of the abovementioned plasmids (pSC4573 and pKPO2) were electroporated into these strains to verify the expressions of glycoproteins. The results showed that glycoprotein expression in WdlO-d01 was significantly higher than that in W3110 ∆*waaL*, but decreased abruptly after further deletion of ECA and CPS (Fig. 1d). It was hypothesized that some genes of unknown functions in the ECA and CPS synthesis gene clusters might be involved in the exogenous OPS synthesis process. Thus, WdlO-d01 was chosen as a chassis cell for the subsequent research.

In addition to the O2 serotype, the *K. pneumoniae* O1 serotype was also the dominant clinically prevalent strain. The glycan structure of the O1 strain is longer and more complex than that of the O2 serotype. The O1 antigenic polysaccharide synthesis gene cluster (*wzm*, *wzt*, *wbbM*, *glf*, *wbbN*, *wbbO*, *wbbY*, *wbbZ*) has only two additional genes compared to that of the O2 gene cluster. Therefore, we added the *wbbY* and *wbbZ* genes into the pKPO2 plasmid and constructed a new plasmid pACYC184-KPO1 (pKPO1) (Additional file 1: Fig. S2) expressing the *K. pneumoniae* O1 serotype polysaccharide. The function of this plasmid was further verified by WB, and the ladder bands could be detected in the higher molecular weight regions after it was electroporated into WdlO-d01/pSC4573 strain (Fig. 1e).

### The glycosyl-engineered *E. coli* chassis required only one plasmid to produce the targeted glycoprotein

**Fig. 2:**
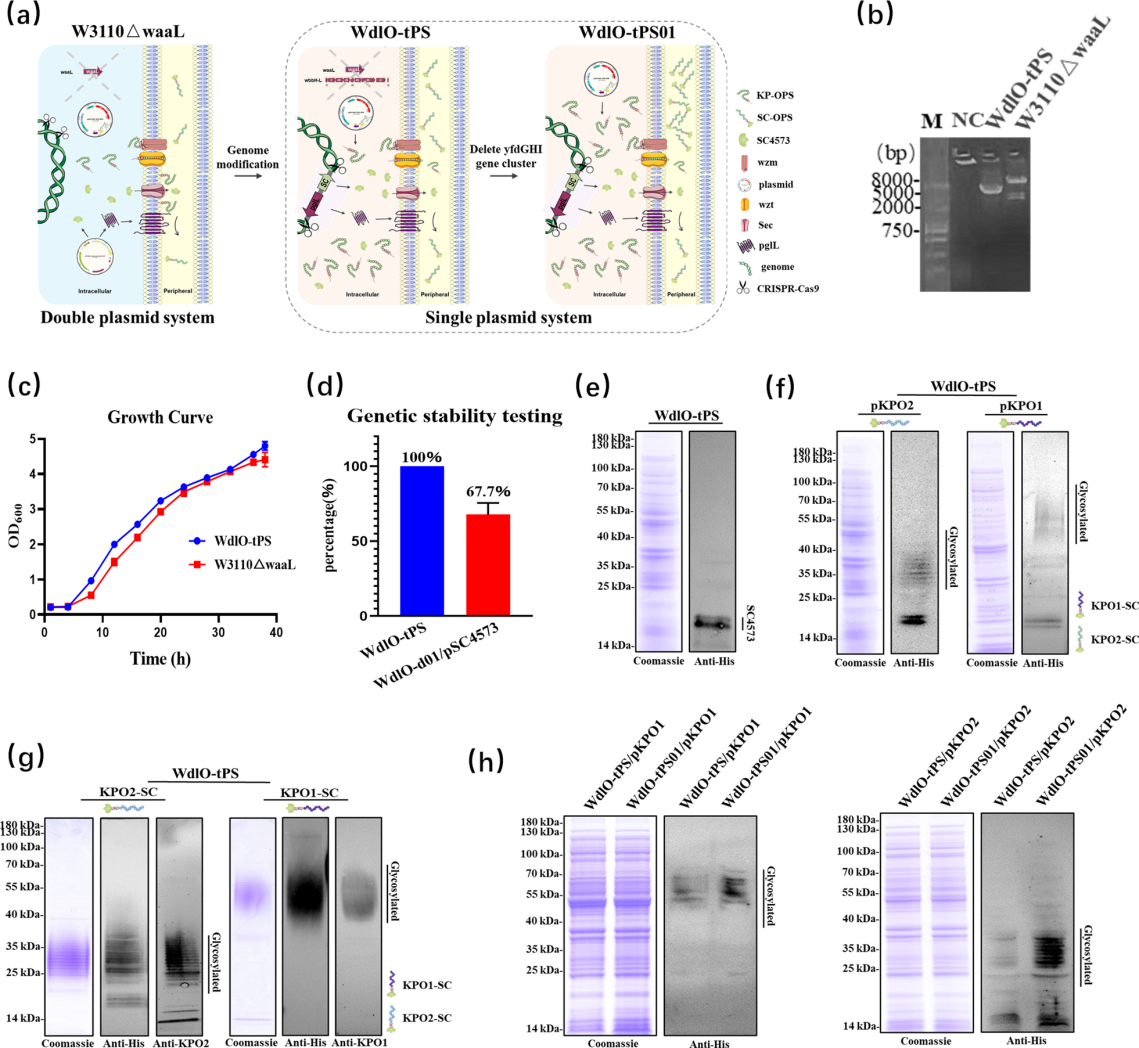
The glycosyl-engineered *E. coli* chassis with genomic integration of acceptor protein SC4573 and glycosyltransferase PglL. **a** Schematic representation of the design of glycosyl-engineered *E. coli* chassis. **b** PCR validation of the expression cassette LacI-pglL-SpyCatcher4573-rrnB. **c** Growth curve of the chassis strain WdlO-tPS. Each strain was inoculated with a seed solution at OD_600_ = 2.0, using an LB ratio of 1:100. **d** Genetic stability testing. After 10 generations of passaging, 96 monoclonal clones were selected and tested for positivity of the *SC4573* and *pglL* genes in strains WdlO-d01/pSC4573 and WdlO-tPS using PCR. These were tested three times, and the positivity of the *SC4573* and *pglL* genes in strains WdlO-d01/pSC4573 were 66.6% (32/96), 60.5% (38/96), 76.1% (23/96), respectively, with a mean of 67.7%. **(e)** Detection of acceptor protein SC4573 expression in strain WdlO-tPS. **f** Detection of KPO1-SC and KPO2-SC glycoprotein expression in strain WdlO-tPS. **g** Purified KPO1-SC and KPO2-SC glycoproteins produced by strain WdlO-tPS were analyzed by SDS-PAGE, and WB with anti-His, anti-KPO1, and ant-KPO2 antibodies. **h** Comparison of KPO1-SC and KPO2-SC glycoprotein expression before and after deletion of the *yfdGHI* gene cluster in strain WdlO-tPS

Encouraged and inspired by the elegant works of Prof. DeLisa’s team [27], we integrated the necessary components (glycosyltransferase PglL and the acceptor protein SC4573) into the genome of *E. coli*, leaving the synthesis gene cluster for the antigenic polysaccharide in a plasmid, so as to facilitate the modification and replacement of the polysaccharide antigens to produce different bioconjugate vaccines. Thus, we redesigned a LacI-pglL-SpyCatcher4573-rrnB expression cassette (consisting of the lactose operon repressor protein LacI, promoter *trc*, O-linked glycosyltransferase PglL, acceptor protein SpyCatcher and glycosyltransferase recognition sequence 4573, and terminator *rrnB*) flanked by the upstream and downstream sequences of the *wbbH–L* gene cluster to integrate into the bacterial genome and replace the *wbbH–L* gene cluster locus by homologous recombination mediated by CRISPR-Cas9 (Fig. 2a). The designed chassis (named WdlO-tPS) was successfully constructed (Fig. 2b), and further verified by PCR and sequencing. There was no significant difference in the growth curve and the number of viable bacteria compared to the parent strain W3110 ∆*waaL* (Fig. 2c and Additional file 1: Fig. S3). An advantage of the WdlO-tPS chassis with glycosylation-relevant components integration at a genomic locus is the better genetic stability compared to plasmid-based systems. Indeed, after 10 generations of propagation without antibiotic stress, there was no loss of the genes encoded SC4573 and PglL for the WdlO-tPS chassis, while only about 67.7% were remained in the classic plasmid-based system (Fig. 2d).

The acceptor protein SC4573 was correctly expressed in the WdlO-tPS chassis after induction by IPTG (final concentration 1 mM), as shown in the WB results (Fig. 2e). Furthermore, when the *K. pneumoniae* antigenic polysaccharide synthesis plasmids pKPO1 and pKPO2 were electroporated into WdlO-tPS chassis, the expression of the targeted glycoproteins KPO1-SC and KPO2-SC was also detected by WB (Fig. 2f). To demonstrate that the glycosyl-engineered strain was suitable for conjugate nanovaccine development, we prepared a large amount of glycoproteins KPO1-SC and KPO2-SC in shake flasks with a total of 12 L of culture medium. After protein purification, the purity of the targeted product was sufficient to be used in the next stage (Fig. 2g). However, the yields of targeted glycoproteins produced by the WdlO-tPS chassis was lower than expected, possibly because there was only one copy of glycosyltransferase PglL in the genome. After careful examination of the polysaccharide metabolism pathway, we found that the O-antigen side chain modification gene cluster *yfdGHI* (*yfdG*, *yfdH*, *yfdI*) was able to transfer glucose from UDP-glucose to undecadienyl pyrophosphate. Presumably, the deletion of this gene cluster would release more undecadienyl pyrophosphate for the synthesis of target glycans. To test this hypothesis, we used the strain WdlO-tPS to clarify the relationship between the *yfdGHI* gene cluster and glycoprotein expression. Using the CRIPSR-Cas9 system, we successfully knocked out the *yfdGHI* gene cluster and obtained strain WdlO-tPS ∆*yfdGHI* (renamed WdlO-tPS01). It showed no significant difference in the growth curve and viable count compared to the parent strain WdlO-tPS (Additional file 1: Figs. S4 and S5). Subsequently, the plasmids pKPO1 and pKPO2 were introduced into this strain to verify the expression of glycoproteins. As expected, the expression of glycoproteins in strain WdlO-tPS01 was significantly higher than that of strain WdlO-tPS (Fig. 2h and Additional file 1: Fig. S6).

### Preparation of conjugate nanovaccines using the glycoproteins produced by glycosyl-engineered chassis

Purified proteins in *E. coli* are susceptible to LPS contamination, so endotoxin removal is an essential, time-consuming, and expensive part of vaccine production. The deletion of genes associated with lipid A, such as *lpxL*, *lpxM*, *pagP*, *lpxP*, and *eptA*, significantly reduces endotoxin production. LpxM is a Kdo2-lauroyl-lipid IVA myristoyl-ACP acyltransferase whose removal has a less structural impact on the Kdo core of lipid A but significantly reduces dendritic cell activation. Therefore, after obtaining the bacterial chassis WdlO-tPS *∆yfdGHI*, we further removed the *lpxM* gene and renamed the resulting strain WdlO-tPS02 (Additional file 1: Fig. S7). The deletion of *lpxM* had no significant effect on the chassis cells in this study, as verified by glycoprotein expression assays (Additional file 1: Fig. S8).

**Fig. 3:**
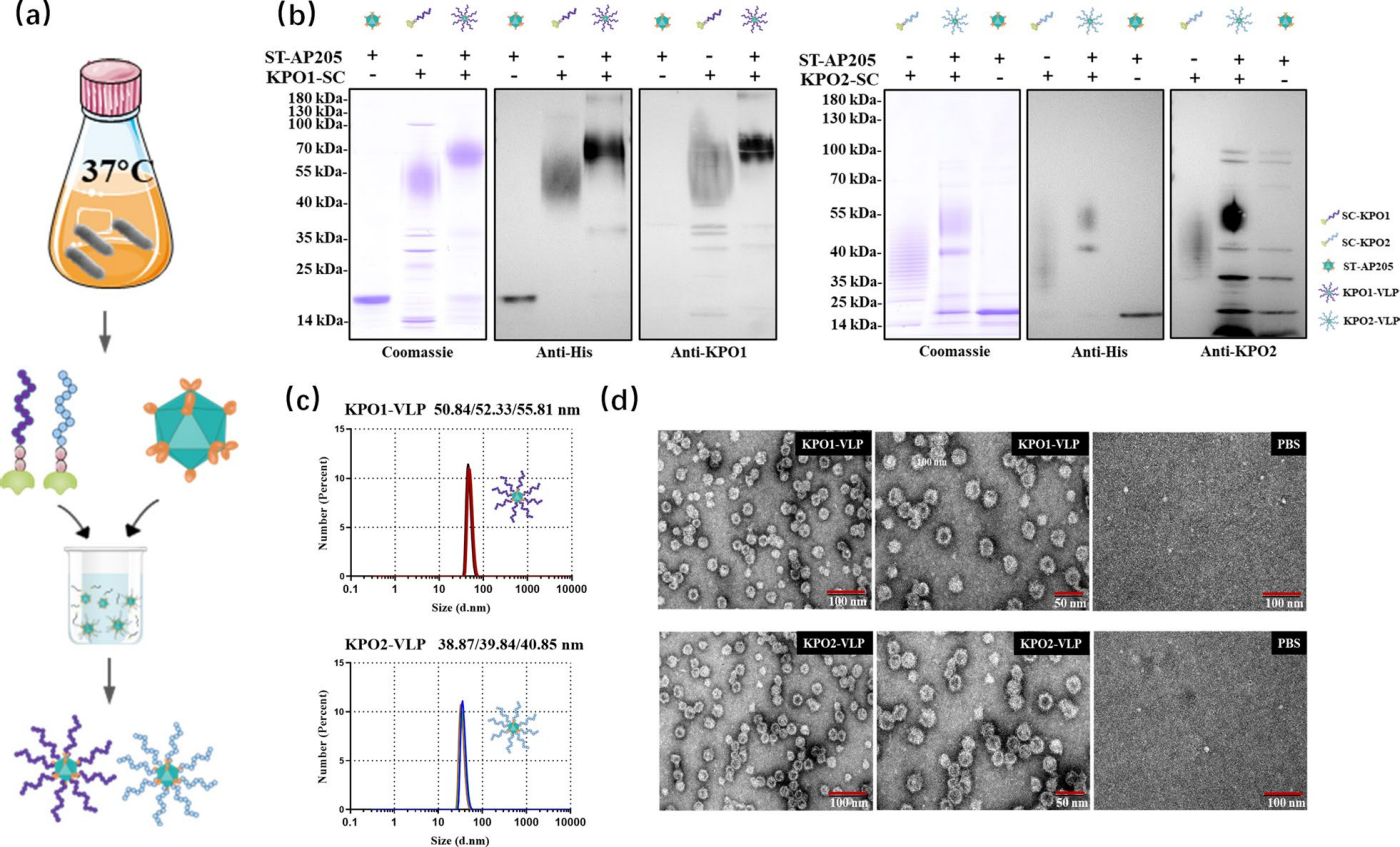
Preparation of conjugate nanovaccines. **a** Schematic diagram of SpyCatcher-SpyTag nanoparticle assembly. **b** SDS-PAGE of KPO1-SC/KPO2-SC bound to ST-AP205 nanoparticles overnight at 4 °C. Anti-His and anti-OPS antibodies were used. **c** Dynamic light scattering (DLS) assay of KPO1-VLP and KPO2-VLP nanoparticles. KPO1-VLP nanoparticle size: 53.0 ± 2.1 nm; KPO2-VLP nanoparticles size: 39.8 ± 0.8 nm. Mean ± standard deviation (SD), n = 3. **d** Transmission electron microscopy (TEM) images of KPO1-VLP and KPO2-VLP nanoparticles. Scale bar: 50 nm or 100 nm

To demonstrate the practical value of the engineered bacteria constructed in this study, we used WdlO-tPS02 as a chassis for *K. pneumoniae* O1 and O2 glycoprotein (KPO1-SC and KPO2-SC) production in shake flasks with a total volume of 12 L of culture medium. High performance liquid chromatography (HPLC) was used to determine the purity of the target proteins. The results showed that purities of KPO1-SC and KPO2-SC were more than 99% and 94%, respectively (Additional file 1: Fig. S9). Then, the purified KPO1-SC and KPO2-SC were combined with SpyTag-Ap205 (ST-AP205) nanoparticles, which was described by our previous work [40], to prepare conjugate nanovaccines. The resulting nanovaccines KPO1-VLP and KPO2-VLP (Figs. 3a and 3b) were obtained after overnight binding in 1 × PBS at 4 °C and removal of unbound proteins by molecular sieving the next day. The particle size of nanovaccines was homogeneous as shown by dynamic light scattering analysis (Fig. 3c) and transmission electron microscopy imaging (Fig. 3d).

### Evaluation of immune effector of conjugate nanovaccines

**Fig. 4:**
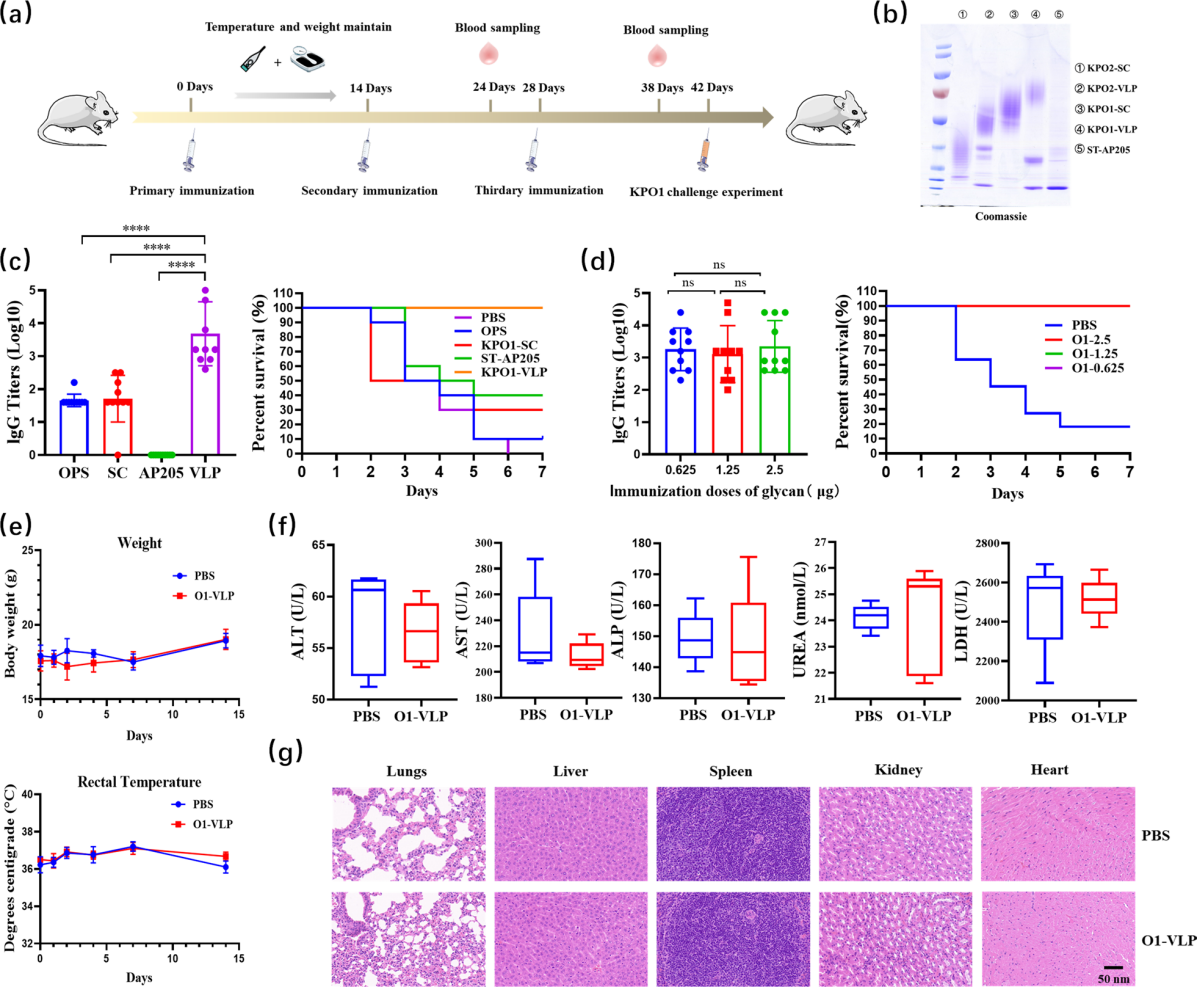
Immunological evaluation of KPO1-VLP in mice. **a** Experimental schedule for the immune assessment. **b** SDS-PAGE assays of KPO2-SC glycoprotein, KPO2-VLP conjugate nanovaccines, KPO1-SC glycoprotein, KPO1-VLP conjugate nanovaccines, and ST-AP205 nanoparticles. **c** ELISA-based measurement of IgG titers against *K. pneumoniae* strain 041 LPS after three subcutaneous immunizations for OPS-KPO1, KPO1-SC, KPO1-VLP, ST-AP205, and sterile 1 × PBS groups of BALB/c mice IgG titers. Fourteen days after the final immunization, 10 mice per group were injected intraperitoneally with *K. pneumoniae* strain 041 (2 × 10^4^ CFU per mouse), and survival was monitored for 7 d. Differences between groups were evaluated using an unpaired one-way analysis of variance (ANOVA) (****p < 0.0001). **d** ELISA-based measurement of IgG titers against *K. pneumoniae* strain 041 LPS after three subcutaneous immunizations of BALB/c mice using KPO1-VLP at different glycan levels with a sterile 1 × PBS group control. Fourteen days after the final immunization, 10 mice in each group were injected intraperitoneally with *K. pneumoniae* strain 041 (2 × 10^4^ CFU per mouse), and survival was monitored for 7 d. Differences between groups were evaluated using unpaired one-way analysis of variance (ANOVA) (ns, differences not statistically significant). **e** BALB/c mice were immunized once subcutaneously using KPO1-VLP (immunization dose of 12.5 μg glycan per mouse) with a sterile 1 × PBS group control; changes in body temperature and body weight were monitored 14 days after immunization. **f** Serum biochemical parameters including alanine aminotransferase (ALT), alanine aminotransferase (ALP), aminotransferase (AST), blood urea nitrogen (BUN), and lactate dehydrogenase (LDH) were monitored 28 days after immunization. **g** The heart, liver, spleen, lungs, and kidneys were isolated for pathological sectioning 28 days after immunization. Scale bar: 50 nm

Because conjugate nanovaccines against *K. pneumoniae* O2 serotype have been investigated by us previously [41], we focused on the immune effects of the nanovaccine against the O1 serotype prepared using our chassis cells in this study. Mice were evaluated after immunization using the conjugate nanovaccine KPO1-VLP (Fig. 4a), and all prepared proteins were grouped for SDS-PAGE validation before immunization (Fig. 4b). Immunological evaluation was performed by randomly grouping 6-week-old mice in 10 animals/group using antigens OPS-KPO1, KPO1-SC, KPO1-VLP (immunization doses of 2.5 μg of glycan per mouse), ST-AP205 (immunization doses of 15.5 μg of protein per mouse, corresponding to 2.5 μg of glycan), and sterile 1 × PBS (control group). Mice were immunized subcutaneously, and serum titers were measured using tail-tip blood collected 10 days after the third immunization and challenged on day 14 using a minimum lethal dose of the *K. pneumoniae* O1 serotype strain 041 [mice were challenged with 2 × 10^4^ CFU (Additional file 1: Fig. S10)]. The results showed that after three immunizations, enzyme-linked immunosorbent assay (ELISA) of IgG titers against *K. pneumoniae* strain 041 LPS were generally between 3 and 4 in the KPO1-VLP group (Log10), between 1 and 2 in the OPS-KPO1 and KPO1-SC groups (Log10), and not detected in the ST-AP205 group (Fig. 4c). When challenged by strain 041, the KPO1-VLP group achieved 100% survival (Fig. 4c). Furthermore, the serum titers did not decrease significantly when the antigenic polysaccharide content was reduced to 1/4, and 100% protection was still achieved (Fig. 4d). The vaccine was also tested for safety in mice at five times the normal immunization dose. The results of biochemical tests and pathological sections of the heart, liver, spleen, lungs, and kidneys showed no significant pathological changes compared to the control group (Fig. 4e–g).

It is generally accepted that the *K. pneumoniae* O2 serotype polysaccharide is less immunogenic due to its simpler glycan chain structure of disaccharide repeating units. In this study, the *K. pneumoniae* O2 conjugate nanovaccine KPO2-VLP was also evaluated in parallel. When challenging mice using the *K. pneumoniae* O2 serotype strain 355 (mice were challenged with 2.5 × 10^7^ CFU per mouse (Additional file 1: Fig. S11)), the O2 conjugate nanovaccine achieved 50% protection and achieved 70% protection after doubling the antigenic polysaccharide amount (data not shown). Similar to the aforementioned O1 serotype results, KPO2-VLP also showed good safety.

## Discussion

To circumvent plasmid instability and reduce the need for antibiotics, we have remodeled the *E. coli* genome for bioconjugate vaccines. Unlike most reported works related to the *N*-glycosyltransferase PglB, the O-glycosyltransferase PglL was integrated into the genome to produce O-linked bioconjugate vaccines in this study. At the same time, the universal recombinant acceptor protein SC4573, which is composed of the commonly used SpyCatcher protein and glycosylation recognition sequence 4573, was also integrated into the genome, allowing the chassis cell to produce the target glycoproteins after incorporating the plasmid expressing the desired glycan. The gene cluster encoding antigenic polysaccharides was not integrated into the bacterial genome in order to facilitate replacement between similar plasmids. Furthermore, all of the nanocarriers (AP205 in this study) with a SpyTag fusion could be used as delivery particles that allowed direct in vitro binding to glycoprotein SC4573-OPS to form different types of conjugate nanovaccines. Thus, in terms of our strategy, the only requirement was to change the plasmid for targeting polysaccharide chain synthesis, which will make the development of future nanovaccines fast and simple. Notably, another O-linked glycosyltransferase PglS from *Acinetobacter baylyi* paired with the acceptor protein ComP has become the only glycosyltransferase so far capable of transferring glycan with glucose at the reducing end [9, 42]. The glycosyl-engineered *E. coli* chassis for the PglS system will further expand the applications of PGCT in the future.

A wide range of genes are involved in bacterial polysaccharide synthesis, including nucleotide sugar precursor synthesis genes, glycosyltransferase genes and polysaccharide processing genes (such as side chain modifications). In glycosyl-engineering studies, in addition to the target exogenous glycan, host cells often express many other glycan structures, and these non-target glycan synthesis pathways might interfere with the synthesis and assembly of target polysaccharide antigens by competing for lipid carriers and activating monosaccharide substrates. Therefore, removing non-essential glycan synthesis gene clusters would facilitate the correct and efficient expression of the target polysaccharide antigens. In this study, we aimed to eliminate unnecessary glycan chain interference by removing endogenous, non-essential polysaccharide synthesis gene clusters such as the enterobacterial common antigen (ECA), the *wbb* gene cluster involved in OPS synthesis, the *galF-gnd* gene cluster involved in CPS synthesis, and the *yfdGHI* gene cluster involved in side-chain modification. A limitation in this study, however, is that while the deletion of the *wbb* and *yfdGHI* gene clusters did increase expression, the deletion of both the ECA and CPS synthesis gene clusters did not achieve the expected increase in yield. ECA synthesis gene clusters generally consist of three classes of genes: monosaccharide synthesis genes (*wzzE*, *wzxE*, and *wzyE*), glycosyltransferase genes (*wecA*, *wecF*, *wecG*), and triose unit processing genes (*wecB*, *wecC*, *rmlB*, *rmlA*, *wecD*, *wecE*) [43]. ECA biosynthesis results from the coordinated action of multiple genes, which are present in clusters, usually between the *wecA* and *wecG* loci. O-antigen polysaccharide synthesis is similarly regulated by the coordinated action of multiple genes, usually located between the *galF* and *gnd* loci of the genome [44]. Some of genes are functionally interchangeable, such as the enzymes RmlA and RmlB that are present in both O-antigen and ECA gene clusters [45]. It is hypothesized that the *wbbH–L* gene cluster contains enzymes that are potentially functionally interchangeable with the ECA gene cluster and that these enzymes are also involved in the expression of the glycoconjugate chain in *K. pneumoniae*, so that the deletion of one gene cluster alone can be compensated for by enzymes expressed in the other gene cluster. However, the deletion of both clusters would block the expression of the target glycan.

The two polysaccharide synthesis gene clusters used in this study are relatively simple in structure and easy to clone. By contrast, most of the glycan synthesis gene clusters of other pathogenic bacteria are generally longer, ranging from a dozen kilobases to even hundreds of kilobases, which makes the complete cloning of polysaccharide gene clusters an urgent problem. An effective solution is to modularize gene clusters into individual clonable components and reassemble these genetic elements using synthetic biological methods through standardized interfaces, such as Golden Gate cloning. In addition, from a synthetic biological perspective, key components of PGCT, such as glycosyltransferase PglL, can also be finely regulated by replacing more efficient promoters or stacking multiple promoters. Furthermore, the use of the tandem glycosylation recognition sequence fusion (up to 9 copies) with acceptor proteins [46] can further increase the glycan-to-protein ratio, resulting in higher antigenic polysaccharide content in the bioconjugate vaccines.

Since all heterologous glycan chain synthesis in this study required the initial transfer of *N*-acetylglucosamine (GlcNAc) to UndP catalyzed by the enzyme WecA, the encoding *wecA* gene was retained herein. The first step in the biosynthesis of *E. coli* native glycans, such as ECA and O-antigens, also required this initial enzyme [47]. Therefore, the deletion of *wecA* eliminated the presence of endogenous interfering glycans and produced a cleaner chassis strain suitable for the synthesis of heterologous glycans that are not dependent on GlcNAc initiation transferase [15, 23]. There have been studies on the deletion of the *wecA* gene [23]. In the future, our chassis design could also adopt this strategy for those antigenic polysaccharides with non-GlcNAc at the reducing end to increase the versatility and expand the glycosyl-engineered strain pool.

## Conclusions

Gene deletions and insertions using precise genome editing techniques are among the important steps in the process of constructing chassis cells. In this study, we used the CRISPR-Cas9 system to perform sequential scarless mutagenesis in *E. coli* strain W3110, and integrated two key components of PGCT (glycosyltransferase and acceptor protein) into the targeted genomic locus. This strategy reduces the use of antibiotics and provides greater flexibility for different types of products due to the ease of replacing the plasmid encoding antigenic polysaccharides. We also found that the deletion of the gene cluster *yfdGHI* further increased the expression of targeted glycoproteins. In addition, the deletion of the *lpxM* gene from the chassis strain further reduced the virulence of the strain and created a safe, stable glycosyl-engineered chassis strain. To demonstrate the practical value of this work, we produced glycoproteins bearing *K. pneumoniae* O1 and O2 serotype antigenic polysaccharides using WdlO-tPS02 chassis cells, and then obtained conjugate nanovaccines KPO1-VLP and KPO2-VLP in vitro. These nanovaccines provided high levels of protection against bacterial infections, approximately 100% (KPO1-VLP) and 70% (KPO2-VLP). Overall, the glycosyl-engineered *E. coli* strains described here are designed and optimized for the development of conjugate nanovaccines, and could be a valuable complement to the current pool of glycoengineered chassis cells. This research strategy is also applicable to other PGCT systems (e.g., PglB and PglS) and is expected to be applied in the future for preparing conjugate vaccines for other anti-infectious diseases.

## Materials and methods

### Strains and plasmids

The strains and plasmids used in this study are listed in Table 1. All strains in this paper were sequentially mutated and genomically modified in *E. coli* strain W3110 using CRISPR gene editing technology. *K. pneumoniae* O1 serotype strain 041 and O2 serotype strain 355, for which the genome sequence has been uploaded to the EBI nucleotides database (https://www.ebi.ac.uk/ena/browser/view/PLM06181), were kindly provided by Prof. Yunsong Yu from Sir Run Run Shaw Hospital in China. All primers are shown in Additional file 2: Table S1. All strains were subjected to PCR validation and sequencing (Additional file 1: Fig. S1). All bacterial strains were cultured in Luria–Bertani (LB, peptone 10 g/L, yeast extract 5 g/L, NaCl 10 g/L) broth or on solid LB medium containing 1.5% agar (antibiotics added as necessary to maintain the plasmid in the long-term: kanamycin 50 μg/mL; ampicillin 50 μg/mL; chloramphenicol 50 μg/mL; and spectinomycin 50 μg/mL).

**Table 1:** *E. coli* strains and plasmids used in this study

Strains and plasmids	Genotype descriptions	References
Strains		
W3110 WT	W3110 wild-type strain	[48]
W3110∆*waaL*	*waaL* gene was knocked out in the W3110 strain	This work
WdlO-d01	*wbbH–L* gene cluster was knocked out in W3110∆*waaL*	This work
WdlO-d02	ECA gene cluster was knocked out in WdlO-d01	This work
WdlO-d03	CPS gene cluster was knocked out in WdlO-d01	This work
WdlO-d04	CPS gene cluster was knocked out in WdlO-d02	This work
WdlO-tPS	W3110 ∆*waaL*∆*wbbH–L*:: lacI-pglL-SC4573-rrnB	This work
WdlO-tPS01	*yfdGHI* gene cluster was knocked out in WdlO-tPS	This work
WdlO-tPS02	*lpxM* gene was knocked out in WdlO-tPS01	This work
Plasmids		
pET28a-SpyCatCher4573	Cloning SpyCatCher4573 gene in pET28a	[40]
pACYC184-355-OPS	Cloning 355-OPS gene cluster in pACYC184	[41]
pACYC184-KPO1	Cloning *wbbY–Z* gene cluster in pACYC184-355-OPS	This work
pCas	The CRISPR system plasmid	[39]
pTargetF	The CRISPR system plasmid	[39]

### CRISPR

The gene mutations were performed through the CRISPR method, which has been reported by Jiang et al*.* [39]. Briefly, the N20 portion of the guide RNA (sgRNA) was designed using the online N20 design tool (http://crispor.tefor.net/), and then the designed N20 was constructed into the constitutively expressed plasmid pTargetF (Addgene 62226) using the NEBridge® Golden Gate Assembly Kit (NEB #E1602). The sequence was verified to be correct by sequencing. To obtain the recombinant template, a sequence 750 bp upstream and downstream of the deletion cluster was selected and linked together by using the NEBridge® Golden Gate Assembly Kit (NEB #E1602). The target strain was prepared by electroporation of the 400 ng pCas plasmid (Addgene 62225). One hour before preparation of the competent cells, 2% arabinose was added into the strain to induce the λ Red recombination system which had been constructed in the plasmid pCas. Then 300 ng of the constructed pTargetF plasmid and 800 ng of the recombinant template DNA fragment were electroporated into the strain. After growing on LB plates overnight, the correct editing of the strain was verified by PCR and sequencing (Additional file 1: Fig. S1). Subsequently, edited colonies were cultured overnight at 30 °C in LB with kanamycin and 1 mM IPTG, then serially diluted and plated on kanamycin plates. Spot plates on kanamycin and spectinomycin plates were incubated overnight at 30 °C to identify colonies that had lost pTarget (no spectinomycin resistance) but retained pCas (kanamycin resistance). At this stage, the strains were either induced with 2% arabinose and again made capable of further mutation, or the pCas was removed from the strains by growing overnight at 42 °C without antibiotics.

### Verification of the mutant strain

The mutant strains were tested and verified by PCR for the target DNA fragments and by sequencing. Generally, the upstream and downstream sequences (200–300 bp away from the homology arm) of the target DNA regions were used for the design of PCR primers. Each single clones was picked and resuspended in 10 µL of sterile ddH_2_O, and 1 µL of bacterial suspension was used as a template for PCR amplification using the designed primers. The PCR products were sent for DNA sequencing. The sequencing results were checked against the theoretical sequences using the software BioEdit. The correct clones were selected and inoculated into kanamycin and spectinomycin LB and cultured overnight at 30 °C at 220 rpm. For the preservation of the mutants, the bacterial suspension was mixed with 50% sterile glycerol in a ratio of 1:1 in a biosafety cabinet and frozen at − 80 °C.

### LPS and OPS preparation

LPS was extracted by the hot-phenol method as follows. The culture was collected, washed with PBS, and then suspended in double-distilled water (ddH_2_O). After freezing and thawing three times, an equal volume of 90% phenol was added and followed by vigorous shaking at 68 °C for 15 min. After centrifugation, the water layer was collected in a new tube. The phenol layer was added to ddH_2_O, and this process was repeated again. After that, the collected water layer was dialyzed into ddH_2_O for at least three days, then DNase (5 µg/mL; Solarbio, Beijing, China), RNase (5 µg/mL; Solarbio), and proteinase K (20 µg/mL; Solarbio) were sequentially added and incubated for 2 h at the optimal temperature of each enzyme. After boiling in a water bath for 10 min, the supernatant was collected as the LPS solution. To prepare OPS, glacial acetic acid was added to the extracted LPS solution with a final concentration of 1% (v/v). After boiling in a water bath for 90 min, the mixture was adjusted to a pH of 7.0 with 1 mol/L NaOH and centrifuged at 40,000 *g* for 5 h. The supernatant was collected as the OPS solution.

### Western blot

Western blotting was performed as described previously [40]. Horseradish peroxidase (HRP)-conjugated 6 × His tag antibody (Abmart Shanghai Co., Ltd., Shanghai, China) was used to detect proteins with a 6 × His tag. If there were more non-specific bands, the 6 × His antibody was incubated for 1 h with *E. coli* W3110 cell lysate prior to detection). The antibodies against *K. pneumoniae* serotype O1 and O2 were obtained by immunizing Japanese white rabbits with *K. pneumoniae* strain 355 and *K. pneumoniae* strain 041 whole bacteria respectively, and were used to detect the glycan fraction of glycoproteins. HRP-conjugated goat anti-rabbit IgG (Transgen Biotech, Inc., Beijing, China) was used as the secondary antibody.

### Glycoprotein preparation

The WdlO-tPS01/pKPO1 or WdlO-tPS01/pKPO2 strain was first inoculated into 5 mL of liquid LB (50 µg/mL chloramphenicol), and cultured at 37 ℃ and 220 rpm overnight. Then, 1 mL of bacterial culture was inoculated into a shake flask with 120 mL of LB and incubated at 37 ℃ and 220 rpm for 6 h. When the OD_600_ reached about 1.0, about 10 mL of the bacterial culture was further transferred into a large shake flask with 1.0 L of LB and cultured at 37 ℃ and 220 rpm. To generate sufficient glycoproteins in one batch, a total of 12 L of culture medium was used in this study. When the OD_600_ reached 0.8 in the large shake flask, 0.6 mL of 1 mol/L IPTG was added to each flask, and cells were cultured at 30 ℃ and 220 rpm for 12 h. The bacteria were collected by centrifugation at 8000 rpm for 8 min and stored at − 20 ℃ for use.

### Protein purification

The induced cells were collected by centrifugation at 8000 rpm for 10 min and then were resuspend with Buffer A (0.5 M NaCl, 20 mM Tris–HCL (pH 8.8), 10 mM imidazole, 1 mL of Tween-20, HCL to adjust the pH to 7.5, and makeup distilled water to 1 L). Then the cells were homogenized by using a high-pressure homogenizer. The supernatant was collected by centrifugation at 8000 rpm for 10 min, and was applied to a nickel column (1.6 × 15 cm^2^) that had been equilibrated with buffer A. After the column was washed with buffer A1, the sample was eluted with 100% buffer B (0.5 M NaCl, 20 mM Tris–HCl (pH 7.5), 500 mM imidazole, 1 mL of Tween-20, HCL to adjust the pH to 7.5, and makeup distilled water to 1 L). The eluent was then separated through the Superdex G200 column (1.6 × 90 cm^2^), which had been equilibrated with 1 × PBS buffer. The collected samples were analyzed by SDS-PAGE.

### Animal immunization

Specific pathogen-free (SPF) female 6-week-old BALB/c mice were purchased from Viton Lever Laboratory Animal Technology (Beijing, China), and experiments were performed at the Barrier Laboratory Animal Center. Mice were randomly divided into groups and immunized subcutaneously on days 0, 14, and 28. Bloods were taken by tail snip 10 days after each immunization and serum was stored at 4 °C.

### ELISA

Briefly, 100 μL of LPS solution (100 μg/mL) was added to each well of a 96-well plate. After incubating at 4 °C overnight, the plate was washed three times with PBST. Then, 200 μL of ELISA blocking solution was added to each well and the plate was incubated at 37 °C for 2 h. After washing and drying, serially diluted serum (100 μL/well) was added to each well and incubated at 37 °C for 1 h. Then the plate was washed three times and dried again. The secondary antibody (1:15,000) was added into each well (100 μL) and incubated at 37 °C for 1 h. After the reaction, the plate was washed and dried. A TMB Kit (CWBio, Beijing, China) was used to initiate a color-producing reaction and measure the absorbance of each well at OD_450_.

### Statistical analysis

All analyses were performed using GraphPad Prism 8.0 statistical software (GraphPad Inc., San Diego, CA, USA). Data were analyzed using a *t* test or one-way ANOVA with Dunn’s multiple comparison test. Results were expressed as means ± SDs. Values of *p* < 0.05 were considered statistically significant (****p < 0.0001, ***p < 0.001, **p < 0.01, and *p < 0.05).

## Supplementary Information

**Additional file 1.** **Fig. S1.** PCR and sequencing validation of each deletion strain. **Fig. S2.** KPO1 plasmid construction. **Fig. S3.** WdlO-tPS and W3110 ∆*waaL* viable bacteria count assay. **Fig. S4.** PCR and sequencing validation after deletion of the *yfdGHI* gene cluster of WdlO-tPS strain. **Fig. S5.** WdlO-tPS01 growth curve and viable bacteria count assay. **Fig. S6.** Comparison of protein expression before and after deletion of the *yfdGHI* gene cluster. **Fig. S7.** PCR and sequencing validation after deletion of the *lpxM* gene cluster of WdlO-tPS01 strain. **Fig. S8.** Verification of deletion of the *lpxM *gene of WdlO-tPS01 strain. **Fig. S9.** Purity of target glycoproteins. **Fig. S10**. Mapping the challenge dose of the O1 strain in BALB/c mice. **Fig. S11.** Mapping the challenge dose of the O2 strain in BALB/c mice.

**Additional file 2. Table S1**. Primers used in this study.

## Data Availability

All data generated or analyzed during this study are included in this published article.
