# Supplementary material for: Preparation of a Klebsiella pneumoniae conjugate nanovaccine using glycol-engineered Escherichia coli
Source: Microb Cell Fact. 2023 May 6;22:95. doi: 10.1186/s12934-023-02099-x (PMC10163571; doi:10.1186/s12934-023-02099-x)
Supplement: Supplementary file 1 — Additional file 1. Fig. S1. PCR and sequencing validation of each deletion strain. Fig. S2. KPO1 plasmid construction. Fig. S3. WdlO-tPS and W3110 ∆waaL viable bacteria count assay. Fig. S4. PCR and sequencing validation after deletion of the yfdGHI gene cluster of WdlO-tPS strain. Fig. S5. WdlO-tPS01 growth curve and viable bacteria count assay. Fig. S6. Comparison of protein expression before and after deletion of the yfdGHI gene cluster. Fig. S7. PCR and sequencing validation after deletion of the lpxM gene cluster of WdlO-tPS01 strain. Fig. S8. Verification of deletion of the lpxM gene of WdlO-tPS01 strain. Fig. S9. Purity of target glycoproteins. Fig. S10. Mapping the challenge dose of the O1 strain in BALB/c mice. Fig. S11. Mapping the challenge dose of the O2 strain in BALB/c mice. [file 12934_2023_2099_MOESM1_ESM.pdf]

# Additional Figure:

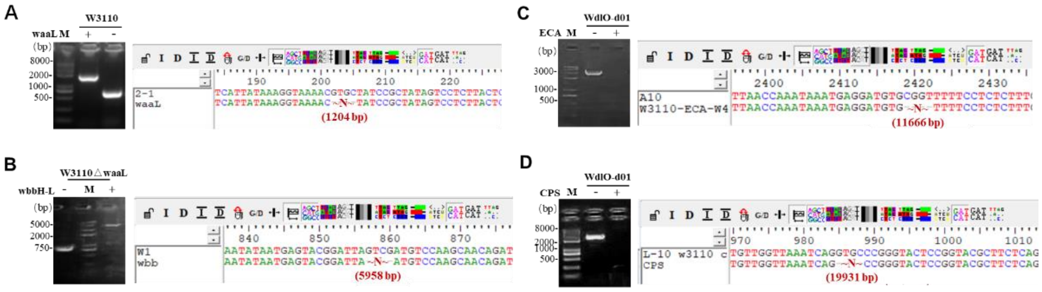


**Figure S1. PCR and sequencing validation of each deletion strain.**

(A) Validation of *waaL* gene deletion.

(B) Validation of the *wbbH–L* gene cluster deletion.

(C) Validation of the ECA gene cluster deletion.

(D) Validation of the CPS gene cluster deletion.

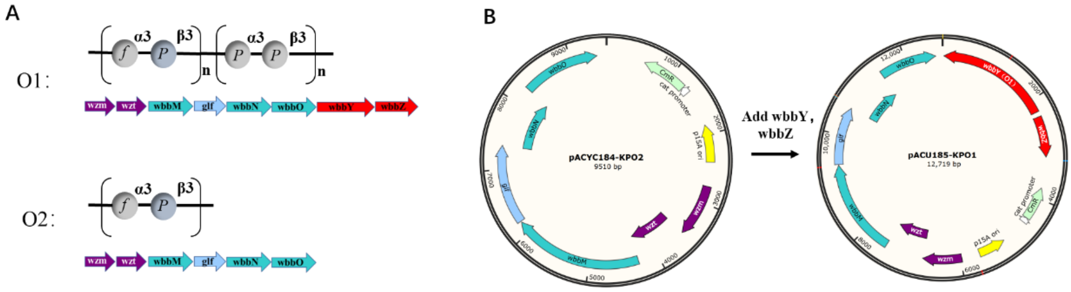


**Figure S2. KPO1 plasmid construction**

(A) Schematic diagram of the structures of *Klebsiella pneumoniae* serotype O1 and O2 glycoforms and glycan clusters.

(B) Schematic diagram of the construction of the *Klebsiella pneumoniae* O1 serotype polysaccharide expression plasmid pACYC184-KPO1.

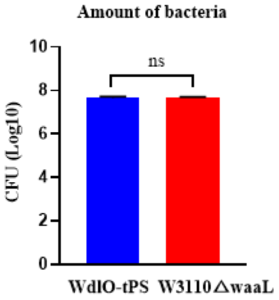


**Figure S3.** WdlO-tPS and W3110 Δ*waaL* viable bacteria count assay. The number of viable bacteria was counted by coating LB plates at gradient dilution at $OD_{600} = 2.0$. Three replicates

per group and differences between groups were evaluated using a *t*-test (ns, differences not statistically significant).

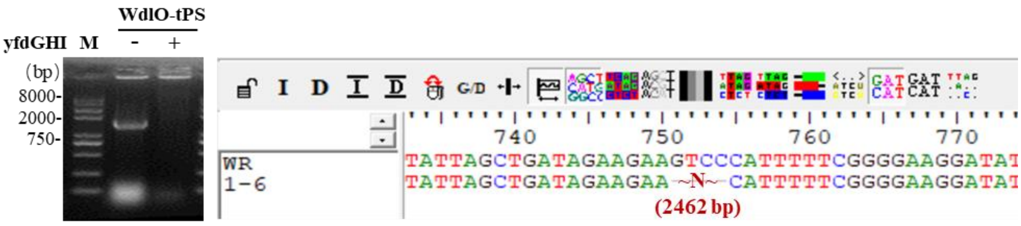


**Figure S4.** PCR and sequencing validation after deletion of the *yfdGHI* gene cluster of WdlO-tPS strain.

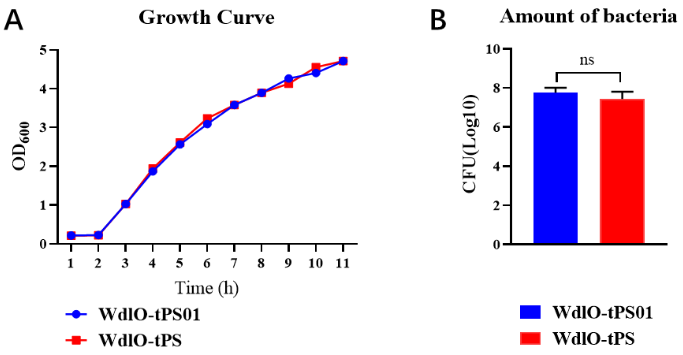


**Figure S5. WdlO-tPS01 growth curve and viable bacteria count assay.**

(A) Growth curve assay for strain WdlO-tPS01. For the growth curve assay, each strain was inoculated with a seed solution at $OD_{600}$ about 2.0, at a seed solution: LB ratio of 1:100.

(B) Strain WdlO-tPS01 viable count assay. The viable bacteria were counted at gradient dilution of coated LB plates at $OD_{600}$ about 2.0. Three replicates per group and differences between groups were evaluated using a *t*-test (ns, differences not statistically significant).

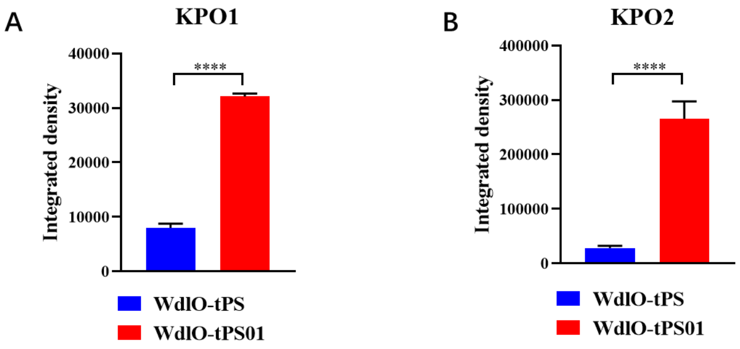


**Figure S6. Comparison of protein expression before and after deletion of the *yfdGHI* gene cluster.**

Protein expression was quantified using the gray scale values of the protein bands on the same

western blot membrane in different lanes (Fig 2h). The results were analyzed using the integrated grayscale density. All groups were required to deduct the background integrated grayscale density.
(A) Comparison of KPO1-SC glycoprotein expression in chassis strains WdlO-tPS and WdlO-tPS01. Three replicates per group and differences between groups were evaluated using a *t*-test (****, $p < 0.0001$).
(B) Comparison of KPO2-SC glycoprotein expression in chassis strains WdlO-tPS and WdlO-tPS01. Three replicates per group and differences between groups were evaluated using a *t*-test (****, $p < 0.0001$)

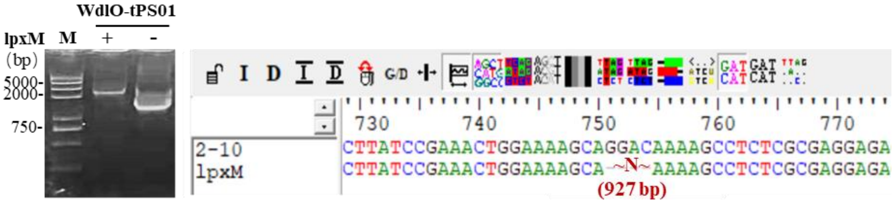


**Figure S7.** PCR and sequencing validation after deletion of the *lpxM* gene cluster of WdlO-tPS01 strain.

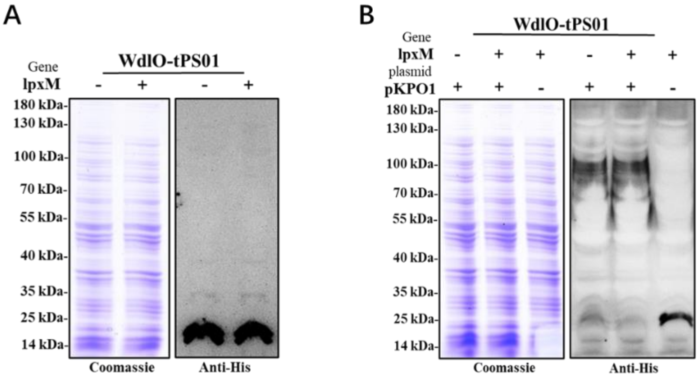


**Figure S8. Verification of deletion of the *lpxM* gene of WdlO-tPS01 strain.**
(A) Detection of SC4573 protein expression following deletion of the *lpxM* gene in chassis strain WdlO-tPS01.
(B) Detection of KPO1-SC glycoprotein expression before and after deletion of the *lpxM* gene in chassis strain WdlO-tPS01.

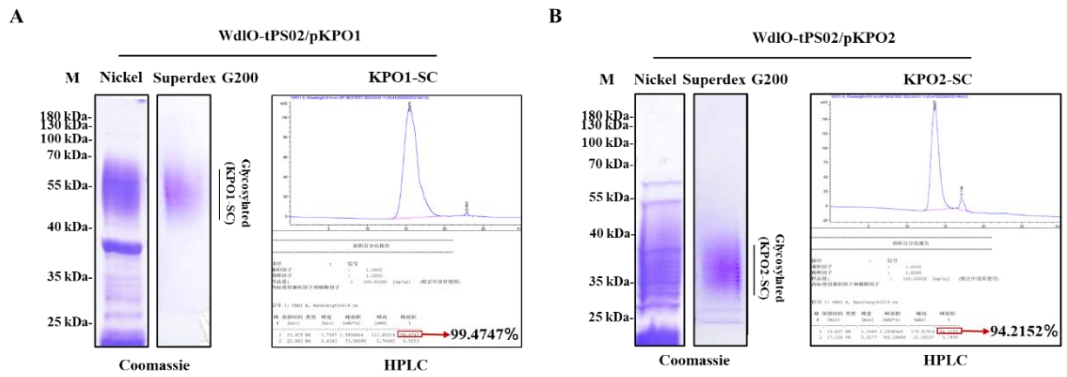


**Figure S9. Purity of target glycoproteins.**

The KPO1-SC and KPO2-SC were first separated by affinity chromatography using nickel resins and further purified by size exclusion chromatography (SEC) using Superdex G200 resins. The purity was detected by high-performance liquid chromatography (HPLC).

(A) KPO1-SC glycoproteins analyzed by SDS-PAGE and HPLC.

(B) KPO2-SC glycoproteins analyzed by SDS-PAGE and HPLC.

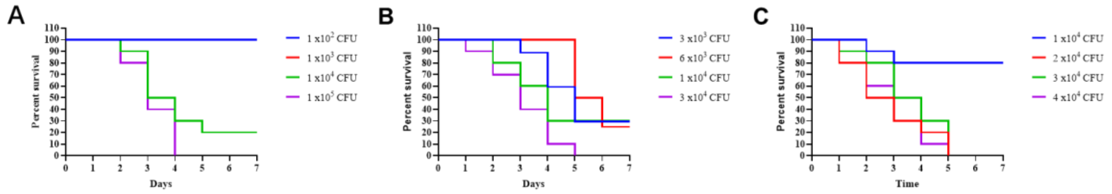


**Figure S10. Mapping the challenge dose in BALB/c mice**

*K. pneumoniae* serotype O1 strain 041 was cultured at $OD_{600}$ about 2.0. Then the bacterial solution was diluted at different concentrations and the BALB/c mice were challenged intraperitoneally

(A) Groups of 10 BALB/c mice were injected with strain 041 at $1 \times 10^2$ CFU per mouse, $1 \times 10^3$ CFU per mouse, $1 \times 10^4$ CFU per mouse, and $1 \times 10^5$ CFU per mouse respectively, and all mice died at the dose of $1 \times 10^5$ CFU.

(B) Groups of 10 BALB/c mice were injected intraperitoneally with strain 041 at doses of $3 \times 10^3$ CFU per mouse, $6 \times 10^3$ CFU per mouse, $1 \times 10^4$ CFU per mouse, and $3 \times 10^4$ CFU per mouse respectively, and all mice died at the dose of $3 \times 10^4$ CFU per mouse.

(C) Groups of 10 BALB/c mice were injected intraperitoneally with strain 041 at doses of $1 \times 10^4$ CFU per mouse, $2 \times 10^4$ CFU per mouse, $3 \times 10^4$ CFU per mouse, and $4 \times 10^4$ CFU per mouse respectively, and all mice died at doses of $2 \times 10^4$ CFU per mouse, $3 \times 10^4$ CFU per mouse, and $4 \times 10^4$ CFU per mouse.

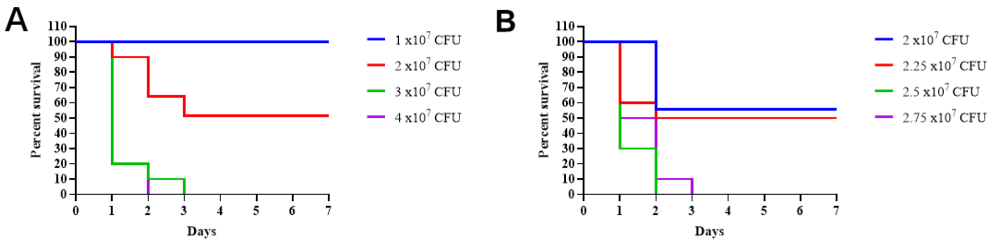

**Figure S11. Mapping the challenge dose in BALB/c mice**

The *K. pneumoniae* O2 serotype strain 355 was cultured at $OD_{600}$ about 2.0. Then the bacterial solution was diluted at different concentrations and the BALB/c mice were challenged intraperitoneally.

(A) Groups of 10 BALB/c mice were injected intraperitoneally with strain 355 at $1 \times 10^7$ CFU per mouse, $2 \times 10^7$ CFU per mouse, $3 \times 10^7$ CFU per mouse, and $4 \times 10^7$ CFU per mouse respectively, and All mice alive at $1 \times 10^7$ CFU per mouse, and all mice died at both doses of $3 \times 10^7$ CFU and $4 \times 10^7$ CFU per mouse.

(B) Groups of 10 BALB/c mice were injected intraperitoneally with strain 355 at $2 \times 10^7$ CFU per mouse, $2.25 \times 10^7$ CFU per mouse, $2.5 \times 10^7$ CFU per mouse, and $2.75 \times 10^7$ CFU per mouse respectively, and all mice died at both doses of $2.5 \times 10^7$ CFU and $2.75 \times 10^7$ CFU per mouse.
